# Pharmacoepidemiology in Japan: medical databases and research achievements

**DOI:** 10.1186/s40780-015-0016-5

**Published:** 2015-05-01

**Authors:** Shiro Tanaka, Kahori Seto, Koji Kawakami

**Affiliations:** Department of Pharmacoepidemiology, Graduate School of Medicine and Public Health, Kyoto University, Yoshida Konoe-cho, Sakyo-ku, Kyoto 606-8501 Japan

**Keywords:** Pharmacoepidemiology, Medical database, Cost-effectiveness

## Abstract

Pharmacoepidemiology involves development of new models to predict safety in the development stages of pharmaceutical products, development of various guidelines and policy related to clinical trials, pharmacovigilance, establishment and implementation of risk management in postmarketing studies, and cost-effectiveness research in medical and social welfare sectors. Evaluations of safety, efficacy, and costs of pharmaceutical products must be developed in a different way. More recently, “big data” in medicine have become the driving force behind epidemiological studies that attempt to solve questions in the clinical setting. Furthermore, it is important to pursue cost-effectiveness considering the government’s financial condition. Epidemiologic and economic research utilizing epidemiological data linked to cost data will provide scientific evidence for appropriate distribution of health resources.

## Introduction

Pharmacoepidemiology is a novel research area established in the 1980s. Its core lies at the intersection of two subspecialties: clinical epidemiology and clinical pharmacology [1]. It covers a wide area including pharmacovigilance, research on the efficacy and safety of drugs [2], regulatory science with assessment of the safety of pharmaceutical products, comparative effectiveness research (CER), cost-effective analysis (CEA), and guideline assessment [3]. Few are experienced in these fields, so more researchers and promotion of these areas are desirable.

The typical focus of pharmacoepidemiology is pharmacovigilance or postmarketing surveillance (PMS), also called phase IV trials. The gold standard for clinical research is randomized clinical trials (RCTs) [4]. However, clinical trials have strict inclusion criteria and rarely include elderly patients, pregnant women, and those with multiple comorbidities. Moreover, the duration is relatively short and the number of subjects are limited [5]. Therefore, we cannot expect these trials to accurately reflect actual drug use in real-life clinical settings. Observational studies with longer follow-up times, inclusion of patients with concomitant illnesses, and larger patient numbers may better identify clinically important adverse events compared with RCTs [6]. It is essential to perform pharmacoepidemiological studies to identify adverse effects and drug efficacy in the real-world setting using a large-scale medical database [7].

## Review

1. Classification of medical databases

The databases that are available for use in pharmacoepidemiological studies include administrative claims database, pharmacy dispensing database, electronic medical records, and care information. In Japan, large-scale medical databases were constructed by the government in the past decade. The Ministry of Health, Labour and Welfare (MHLW) developed a national claims and health checkup database (NDB) and the Sentinel project to detect adverse drug reactions based on the medical records of large hospitals [8,9]. In addition, the Japan Surgical Society developed the National Clinical Database (NCD) as a patient registry to identify drug efficacy and outcomes [10].

2. Medical databases in Japan

### Claims database

The largest claims database available for academic and industry use is provided by the Japanese Medical Data Center (JMDC), Co. [11]. The JMDC collects claims data from approximately 2,300,000 people who belong to a health insurance provider for company employees. It includes data from inpatients, outpatients, and pharmacy claims as well as Diagnosis Procedure Combination (DPC) data collected every month, and all items listed together are placed into the database. Secondary data are not only utilized by the health insurance union but also by pharmaceutical companies with secondary use permission, thereby contributing to epidemiological studies [12]. The advantage of the claims database is that it can provide diagnosis and treatment information even if a patient switches to another clinic or hospital, but it cannot provide examination results or clinical outcomes.

The DPC is a Japanese case-mix classification system launched in 2002 by the MHLW and is linked to a lump-sum payment system. Approximately 1,500 hospitals use DPC in acute medical care, and a database based on DPC is provided by Medical Data Vision (MDV), Co., covering 143 facilities that represent approximately 10% of institutions in Japan. This database is an electronic health records-based database that contains anonymous information from health insurance claims of about 8.5 million patients from April 2008 to 2014. It contains patient information such as age, sex, relevant medical department, disease name on the prescription, and information on medications, surgery, injections, tests, diagnosis in DPC claims, patient outcomes, and results of blood tests and other laboratory tests [13]. Detailed treatment history is included in the DPC data in a particular hospital.

### Pharmacy dispensing database

A few Japanese companies that run pharmacies such as Nihon Chouzai Co. also maintain their own administrative database, which includes information on dispensing drug formulas. Our research group combines data from four companies that own pharmacies to analyze approximately 20 million prescriptions per year. This dataset enables us to identify patterns in drug prescriptions nationwide and patient adherence to drugs [14].

### Other hospital databases

The Platform for Clinical Information Statistical Analysis (CISA) is another medical database that collects claims data from 14 Japanese national university hospitals. Anonymous medical records in each institution are integrated and analyzed. Establishing a linkage with laboratory results data is currently underway. A pharmacoepidemiological study of the patterns of prescription of osteoporosis drugs for patients on glucocorticoids or those with lifestyle-related disease-related in Japan has been performed utilizing the CISA database [15].

### Methodological and ethical considerations

Because each database has different characteristics in terms of clinical and administrative records, researchers performing pharmacoepidemiological studies need to clearly recognize the strengths and weaknesses of each database [16]. According to the Ethical Guidelines on Biomedical Research Involving Human Subjects by Ministry of Education, Culture, Sports, Science and Technology and Ministry of Health, Labour and Welfare [17], pharmacoepidemiological studies of medical databases would be classified as research based on pre-existing material and information without any invasions and interventions. The guidelines require a study protocol which includes design and conduct of the study as well as ethical issues such as handling of privacy information, disclosure of the study and conflict of interest, but written informed consent is not mandatory for studies of medical databases.

3. Pharmacoepidemiological studies in Japan

### Drug safety study

The first example of a pharmacoepidemiological study in Japan is a drug safety study of palivizumab, an anti-respiratory syncytial virus humanized monoclonal antibody used for prophylaxis against severe lower respiratory tract infection in children [18]. It was difficult to conduct a comparative study in Japan because the clinical guideline, universal health insurance system, and subsidy for palivizumab permit prescription of the drug to almost all patients who need it. The researchers therefore studied the adverse events of palivizumab by applying a novel study design called the self-controlled case series method. The aims of the study were to clarify the advantages and difficulties of the self-controlled case series method compared with cohort studies, and to explore the impact of different definitions of periods and events used for the analysis on the results. The database used in this example consisted of anonymized records from 16 DPC hospitals in Japan provided by MDV. A total of 70,771 eligible children from neonates to those aged 5 years were identified and 641 patients were treated by palivizumab. The incidence rate ratios for diarrhea, bronchitis, and eczema were 3.0 (95% confidence interval [CI] 1.7–5.4), 10.3 (95% CI 8.0–13.2), and 16.9 (95% CI 12–23), respectively, but results varied greatly depending on the definitions of the periods and events used for the analysis. The researchers concluded that the self-controlled case series method using administrative databases could be a useful tool in pharmacoepidemiological studies of children, but these studies should be viewed as hypothesis-generating rather than confirmatory.

### Drug utilization study using a claims database

Administrative databases also provide an important means to describe prescription patterns [12] and utilization trends [unpublished data]. Katada et al. investigated the prescription patterns and trends for antirheumatic drug use in Japanese patients with rheumatoid arthritis (RA) and examined whether these patients are being treated according to EULAR recommendations and ACR guidelines [12]. The researchers used a large-scale claims database managed by JMDC containing the claims of employee health insurance recipients [1,11] to identify 5,126 users of antirheumatic drugs with diagnosis codes of RA. The number of patients who received disease-modifying antirheumatic drugs (DMARDs) including biologics as initial therapy was 629 (12.3%), while the others received non-DMARD therapy only, although the guidelines in the USA and Europe recommend aggressive first-line treatment. During the study period, the use of methotrexate and biologics as first-line drugs increased from 1.9% to 8.0% and from zero to 1.6%, respectively (p < 0.001 for both), while the use of non-steroidal anti-inflammatory drugs decreased (p = 0.004). These findings suggest that many early RA patients in Japan do not receive aggressive treatment, although this prescribing practice is gradually changing to better comply with clinical recommendations.

### Drug utilization study using pharmacy dispensing databases

Cancer care in Japan has rapidly changed from in-hospital care to outpatient care and from in-hospital prescription to external prescription at pharmacies. Therefore, Takizawa et al. studied the use of oral anticancer medicines in pharmacies that accept insurance [unpublished data]. This study analyzed the dispensing databases of 489 pharmacies that are managed by two major pharmacy chains in Japan. A total of 31,628 patients who received oral anticancer medicines between June 1, 2011 and May 31, 2012 with 156,904 prescriptions were identified in the databases. These patients received hormone therapy (n = 19,899 [62.9%]), anti-metabolic medicines (n = 9,002 [28.5%]), molecularly targeted medicines (n = 1716 [5.4%]), alkylating compound medicines (n = 839 [2.7%]), microtubule inhibitors (n = 148 [0.5%]), and immune-suppressing agents (n = 24 [0.1%]). These findings suggest not only increasing use of oral anticancer medicines in pharmacies that accept insurance but also the importance of pharmacy–clinic cooperation in clinical practice. Patients in the pharmacy dispensing dataset are unique because they are all outpatients, but the data may include uninsured dispensing, unlike the claims databases.

4. Health technology assessment and pharmacoepidemiology

Health technology assessment (HTA) is a method of evidence synthesis that considers evidence regarding clinical effectiveness, safety, and cost-effectiveness, and when the term is considered in a broad sense it includes social, ethical, and legal aspects of the use of health technologies [19]. There are processes such as evidence-based medicine (EBM), cost-benefit analysis (CBA), and CER in HTA. In the practice of EBM, HTA considers evidence regarding the efficacy and effectiveness of interventions as well as patient values and is mainly concerned with individual patients’ decisions, but it is also useful for developing clinical guidelines for individual patients. Analyzing the total cost of medical intervention and its benefit depends on CBA. For example, suppose that medical treatments A and B for a specific disease both have a constant curative effect, statistically analyzing the effectiveness of both from the perspective of costs constitutes CER. There are techniques such as Markov models for use in epidemiologic studies. In evaluating anticancer treatment, two indices may be used for CER: quality-adjusted life year (QALY) related to quality of life for the duration of survival of the patient and additional expenses to obtain one more QALY (incremental cost-effectiveness ratio [ICER]) from conventional therapy. A major use of HTA should include benefit–harm assessment and economic evaluation. Pharmacoepidemiology plays an important role in assessing the cost-effectiveness of pharmaceutical products.

The efficacy of statin for people without cardiovascular disease has been established in clinical trials. Nonetheless, it is unclear for whom and when statin treatment should be initiated with regard to absolute risk reduction of cardiovascular disease and cost-effectiveness of long-term statin therapy. Onishi et al. [20] performed cost-effectiveness analysis of statin therapy in populations with different risk factors. The incidence of acute myocardial infarction was estimated using a risk prediction formula derived from a cohort study in Japan, the Japan Arteriosclerosis Longitudinal Study–Existing Cohorts Combined (JALS-ECC). The incremental cost-effectiveness ratios of pravastatin therapy compared with no-drug therapy over a lifetime were 9,677,000 yen per QALY for 55-year-old men and 8,648,000 yen per QALY for 65-year-old men with cardiac risk factors of diabetes mellitus, hypertension (grade II), and smoking, and statin therapy was not cost-effective compared with no-drug therapy in all evaluated subgroups.

## Conclusions

Pharmacoepidemiological studies utilizing various databases contribute to the medical community by improving medical services and drug safety; assist the industry in identifying unmet needs; aid regulatory agencies in their assessment of efficacy and safety profile of drugs; and help stakeholders including healthcare providers to analyze the cost-effectiveness of drugs. The guideline for pharmacoepidemiological research was adopted by the Pharmaceuticals and Medical Devices Agency in 2014 [21]. This guideline is intended to assist not only investigators of pharmaceutical companies with issues pertaining to the planning, conduct, and evaluation of pharmacoepidemiological studies, but also academic researchers by promoting careful study design with appropriate utilization of medical databases. Establishment of medical databases for the purpose of epidemiological research will be useful, and robust investigations in a variety of therapeutic areas will expand the frontiers of pharmacoepidemiology.

## Abbreviations

CER: Comparative effectiveness research
CEA: Cost-effective analysis
PMS: Postmarketing surveillance
RCTs: Randomized clinical trials
NDB: National Claim insurance and health checkup database
NCD: National Clinical Database
JMDC: Japanese Medical Data Center
DPC: Diagnosis Procedure Combination
CISA: Clinical Information Statistical Analysis
RA: Rheumatoid arthritis
DMARDs: Disease-modifying antirheumatic drugs
MTX: Methotrexate
NSAIDs: Non-steroidal anti-inflammatory drugs
HTA: Health technology assessment
EBM: Evidence-based medicine
CBA: Cost-benefit analysis
QOL: Quality of life
QALY: Quality-adjusted life year
ICER: Incremental cost-effectiveness ratio
